# Confidence amongst Multidisciplinary Professionals in Managing Paediatric Rheumatic Disease in Australia

**DOI:** 10.1155/2018/7807490

**Published:** 2018-01-18

**Authors:** Samuel Cassidy, Andrea Coda, Kerry West, Gordon Hendry, Debra Grech, Julie Jones, Fiona Hawke, Davinder Singh-Grewal

**Affiliations:** ^1^School of Health Sciences, Faculty of Health and Medicine, The University of Newcastle, Health Precinct, No. BE154, P.O. Box 127, Ourimbah, NSW 2258, Australia; ^2^Physiotherapy, The Westmead Children's Hospital, Hawkesbury Rd & Hainsworth Street, Westmead, NSW 2145, Australia; ^3^Institute for Applied Health Research, School of Health & Life Sciences, Glasgow Caledonian University, Cowcaddens Road, Glasgow G4 0BA, UK; ^4^Sydney Children's Hospital (SCH), High St, Randwick, NSW 2031, Australia; ^5^Honorary Associate Discipline of Paediatrics and Child Health, The University of Sydney, Sydney, NSW 2006, Australia; ^6^School of Paediatrics and Child Health, The University of Sydney and Department of Rheumatology, The Sydney Children's Hospitals Network (Westmead and Randwick), Sydney, NSW, Australia

## Abstract

**Objective:**

Interprofessional collaboration is a crucial component of care for children with rheumatic disease. Interprofessional care, when delivered appropriately, prevents disability and improves long-term prognosis in this vulnerable group.

**Methods:**

The aim of this survey was to explore allied health professionals' and nurses' confidence in treating paediatric rheumatology patients.

**Results:**

Overall, 117 participants were recruited, 77.9% of participants reported being “not confident at all,” “not confident,” or “neutral” in treating children with rheumatic diseases (RD) despite 65.1% of participants reporting having treated >1 paediatric rheumatology case in the past month. Furthermore, 67.2% of participants felt their undergraduate education in paediatric rheumatology was inadequate. “Journals” or “texts books” were used by 49.3% of participants as their primary source of continuing professional development (CPD) and 39.3% of participants indicated that they did not undertake any CPD related to paediatric rheumatology. Small group and online education were perceived to be potentially of “great benefit” for CPD.

**Conclusion:**

This paper highlights allied health professionals' and nurses' perceived inadequacy of their undergraduate education in paediatric RD and their low confidence in recognising and treating RD. Undergraduate and postgraduate education opportunities focusing on interprofessional collaboration should be developed to address this workforce deficiency.

## 1. Introduction

Rheumatic disease (RD) is an important cause of acquired disability in children [1]. RD in children often results in poorer outcomes than similar conditions in adults, due to greater disease severity and a longer duration of disease over a lifetime [2]. Juvenile idiopathic arthritis (JIA) is the most prevalent paediatric RD [2, 3]. Other less frequent but more severe inflammatory RD of childhood include juvenile systemic lupus erythematosus (SLE), juvenile dermatomyositis (JDM), vasculitis, and scleroderma [1].

Interprofessional care is underpinned by adequate training of health professionals within their own discipline and in their role within the interprofessional team. Any deficiency will flow through the health care team and limit the achievable outcomes for their patients [3–7]. Continuing professional development (CPD) is key to ensure competent ongoing care and to reduce the delay in dissemination and implementation of current best evidence [2]. Adequate undergraduate education is required to understand the physiology of the musculoskeletal system (MSK) and pathophysiology of RD. Field specific CPD is essential for health professionals working in the field of RD to ensure the appropriate recognition, treatment, and prompt referral of patients with RD to prevent long-term damage and disability [3, 6, 8]. The aims of this study were to explore (1) allied health professionals' and nurses' confidence in the treatment of paediatric RD; (2) the perceived adequacy of their undergraduate and postgraduate training in paediatric RD; and (3) the perceived utility of a number of forms of educational opportunities.

## 2. Methods

We surveyed a convenience sample of registered allied health professionals and nurses between April and September 2013 who were involved with the treatment and/or education of patients with RD or had the potential to be exposed to children with RD in Australia. Participants were recruited by email through the “Australian Physiotherapy Association,” “Occupational Therapy Australia,” “Rheumatology Health Professionals Association,” “The Australian Psychological Society,” “Australian Podiatry Association,” and “The Allied To Kids Newsletter.” Participants completed the survey online via Survey-Monkey™. All participants consented to participate in accordance with the ethics approval obtained from the Sydney Children's Hospitals Network Research Ethics Committees. Anonymity and confidentiality were maintained for all participants throughout the survey.

The survey was formulated and designed by the authors who are expert allied health, nursing, and medical professionals working in the field of paediatric RD. The survey covered number of years of work in the profession (0–5/6–10/11–15/>15 years); state of practice within Australia; area of work (urban/rural/remote); health care setting (public/private); patient mix (<18 years/adult/mixed); exposure to paediatric rheumatic conditions; conditions treated; number of children with RD treated in the last month; confidence working with children with rheumatic diseases (rated on a Likert scale from ‘not confident at all' to “very confident”); specific undergraduate education/training (didactic teaching/practical/other); perceived adequacy of undergraduate education (rated on a Likert scale from “strongly-agree” to “strongly-disagree”); participation in formal postgraduate education in paediatric rheumatology; if no CPD, why; and the forms of education that would facilitate skills in managing paediatric RD (increased undergraduate education/improved postgraduate exposure/greater content at professional conferences/access to seminars and workshops/online based education/clinical attachments). Descriptive statistics were used with Excel (Microsoft™) to summarise the data.

## 3. Results

One hundred and seventeen participants consented to participate and completed this preliminary survey investigation. The distribution across health professions is shown in Table 1. Occupational therapists and physiotherapists together made up more than half of the respondents (54.7%; 64/117). Overall, years working in the profession ranged from the categories 0–5 years to >15 years.

A majority of participants were from NSW (New South Wales) (68.3%; 80/117). Respondents were predominantly located in urban areas (72.4%; 79/109) in the public health setting (88.1%; 97/110). Of the respondents, 66% (75/114) saw only children < 18 years of age and 21.9% (25/114) worked in a mixed setting. Sixty-two percent (71/114) had managed paediatric RD currently or previously. Of these, 92.9% (66/71) had done so over the preceding month. When asked how many cases of RD they currently managed, 36.4% (24/66) treated 1-2 cases, 28.8% (19/66) treated > 2, and 9.1% (6/66) saw > 10 cases in the preceding month. JIA and hypermobility were the most frequent conditions managed (Table 2).

Forty-two percent (45/107) reported to be “not confident at all” or “not confident” in treating children with RD. Only 28.1% (30/107) self-evaluated as being “very confident” or “somewhat confident” in treating paediatric RD.

The majority of participants (67.3%; 72/107) “disagreed” or “strongly disagreed” that their undergraduate paediatric RD education was adequate. Fifty-six percent (60/107) reported having received no formal undergraduate paediatric rheumatology (PR) education and 35.5% (38/107) reported having received some didactic teaching but no practical exposure.

CPD for PR was predominantly from journal articles or textbooks (49.5%; 53/107), with 42 (39.3%) participants having undertaken no CPD, due to perceived lack of clinical need (75.6%, 31/41), few educational opportunities (31.7%; 13/41), and geographical challenges (17.1%; 7/41). Overall rates of attendance to conferences (16.8%; 18/107), study days (7.5%; 8/107), seminars/workshops (18.7%; 20/107), or in-house education (20.6%; 22/107) was poor, with online education favoured by 25.2% (27/107).

Participants agree that access to seminars, workshops, and skills days (52.3%; 53/107) as well as online and web-based education (49.5%; 52/105) would be of “great benefit.” Other forms of education perceived to be of “some benefit” included increased undergraduate education, improved postgraduate exposure, and professional conferences.

Implementation of a program that allocated an experienced clinical mentor for clinicians in rural and remote areas was suggested as an approach to improving postgraduate CPD. Increasing the accessibility and dissemination of current CPD or new web-based material in rural areas had been suggested as alternative approach to address poor accessibility to postgraduate CPD in RD.

Results indicated that there is a necessity for health professionals to improve their confidence in treatment and management of RD through their own ongoing professional training in order to function well in a multidisciplinary paediatric rheumatic environment.

## 4. Discussion

Interprofessional management teams are the expected standard of care for paediatric RD [3, 8, 9]. To achieve this, health professionals need to be well-educated and confident in managing such disease within their own discipline and aware of the capabilities of other disciplines. Our survey has shown that the majority of participants perceived that their undergraduate education in paediatric rheumatology was inadequate and many reported that they were not confident in managing these conditions. These findings are concerning in the context that majority of respondents worked in environments that might expose them to children with these conditions. More than half of participants in our survey reported not receiving any formal paediatric rheumatology education as an undergraduate. This finding, while at high risk of recall bias, is important as it is the perception of practising health professionals. If the finding does reflect an absence of paediatric RD undergraduate it may be due to the rarity of paediatric RD [2]. Because of the limited exposure of health professionals to paediatric RD comprehensive undergraduate education maybe considered by some to be unnecessary. Health professionals exposed to paediatric RD should access specific CPD in the field of paediatric RD.

This deficiency in education and confidence in managing RD may contribute to the well documented delays in diagnosis of JIA in Australia [5]. For those who did, didactic teaching was the predominant form of exposure. Improved education in, and exposure to, paediatric rheumatology for allied health and nursing students may help avoid these delays and improve outcomes.

Low uptake of CPD in paediatric rheumatology was also identified and the participants reported that increased accessibility to small group seminars, workshops, and skills days as well as online education would be of significant benefit. Participants highlighted the potential for interprofessional web-based interaction to improve patient care.

Freely available online applications and resources such as pGALS [10] may help practitioners who do not commonly consult with paediatric RD cases. pGALS guides a basic assessment of the MSK system in paediatric patients and offers explanations of both normal and abnormal presentations. Paediatric Musculoskeletal Matters (PMM) aims to raise awareness, knowledge, and clinical skills to promote early diagnosis and referral to specialists as part of the multidisciplinary paediatric rheumatology team. This comprehensive online platform is targeted at the level of graduating doctors, and it includes tools, such as the pGALS, to help clinicians examining children's joints [11]. Guided resources such as pGALS have the ability to provide support to experienced, and perhaps more importantly, to inexperienced allied health professionals who may come into contact with paediatric RD cases.

The convenience sampling used for this survey limits the generalisability of the findings. In this survey, a high proportion of respondents reported treating paediatric RD. A random sampling technique would have provided a true cross section and may have found a lesser proportion of nurses and allied health professionals treating paediatric RD but was not undertaken due to inadequate resources. Due to the design of the web-based survey, results were combined for all nurse and allied health divisions. While this provides a greater sample size, it creates limitations due to the heterogeneity of the group. Despite this limitation, those nurses and allied health professionals treating RD are in a position to identify gaps in knowledge and training and to point to preferred education options to address these. To our knowledge, this is the first survey in Australia and internationally to investigate allied health professionals' and nurses' confidence in treating paediatric RD and their perceived adequacy of their undergraduate and postgraduate training in paediatric RD.

## 5. Conclusion

Many allied health professionals and nurses lack confidence in treating paediatric RD cases and felt that undergraduate and postgraduate education in paediatric RD was inadequate. Most participants felt that an increase in accessibility to online and seminars/workshop opportunities would be of great benefit with many other forms of education to be of some benefit. In particular, web-based interaction was identified as an opportunity to improve interprofessional teamwork and patient care. Education opportunities for allied health professionals and nurses in paediatric rheumatology must be improved to achieve early recognition and referral and effective management of paediatric RD. Without improvement children with rheumatic disease will continue to face unnecessary and avoidable delays in receiving correct diagnosis and effective treatment.

## Figures and Tables

**Table 1:** Health professional disciplines and commonly managed paediatric rheumatological conditions.

	Number of respondents (*n*)	Percentage of respondents (%)
Occupational therapist	35	30.7
Physiotherapist	29	25.4
Nurse	13	11.4
Podiatrist	10	8.8
Psychologist	9	7.9
Dietician	7	6.1
Social worker	4	3.5
Diversional therapist	1	0.9
Other^*∗*^	6	5.3

^*∗*^4 speech pathologists, 1 genetic counsellor, and 1 pharmacist.

**Table 2:** Number of respondents who are routinely involved in the management of paediatric rheumatology conditions.

	Number of respondents (*n*)	Percentage of respondents (%)
Juvenile Idiopathic Arthritis	56	47.9
Hypermobility	54	46.2
Pain Syndromes	39	33.3
Mixed Connective Tissue Disorder	34	29.1
Systemic Lupus Erythematosus	18	15.4
Juvenile Dermatomyositis	18	15.4
Scleroderma	16	13.7
Vasculitis	10	8.5

## Additional Points

*(1) What Is Known about the Topic?* Health Professionals (HP) and nurses require adequate education to recognise and treat paediatric rheumatic diseases (RD), including Juvenile Idiopathic Arthritis, to prevent long-term damage and disability.* (2) What Does This Paper Add*? This paper highlights the self-perceived inadequacy of HPs' and nurses' undergraduate education in paediatric RD. Only 28.1% of Australian's HPs and nurses felt “very confident” or “somewhat confident” in treating paediatric RD.* (3) What Are the Implications for Practitioners?* Increased accessibility to seminars, workshops, and skills days as well as online education was reported to be of potential benefit by this group.
